# Lethal/sublethal responses of *Daphnia magna* to acute norfloxacin contamination and changes in phytoplankton-zooplankton interactions induced by this antibiotic

**DOI:** 10.1038/srep40385

**Published:** 2017-01-12

**Authors:** Ying Pan, Shi-wei Yan, Ruo-zhu Li, Yi-wen Hu, Xue-xiu Chang

**Affiliations:** 1 School of Ecology and Environmental Sciences, Yunnan University, Kunming 650091, China; 2 Department of Biology, Yunnan University, Kunming 650091, China

## Abstract

Although the well-known antibiotic norfloxacin (NOR) is recognized as an important environmental pollutant, little is known about its impacts on ecological processes, particularly on species interactions. In this paper, we quantified *Daphnia magna* (Crustacea, Cladocera) responses in mortality rate at lethal NOR concentrations (0, 25, 50, 100, 200, 300 and 400 mg L^−1^), and in heartbeat rate, swimming behavior and feeding rate (on the green alga *Chlorella pyrenoidosa*) at sublethal NOR concentrations (0, 25, 50 and 100 mg L^−1^) to determine the effects of this antibiotic in plankton systems. In 96-h-long lethal experiment, mortality rates of *D. magna* increased significantly with increasing NOR concentration and exposure time. In sublethal experiments, heartbeat rate decreased, while time ratio of vertical to horizontal swimming (TVH) and the duration of quiescence increased in *D. magna* individuals exposed to increasing NOR concentrations after 4 and 12 h of exposure. These collectively led to decreases in both average swimming ability and feeding rate, consistent with the positive relationship between average swimming ability and feeding rate. Overall, results indicate that, by affecting zooplankton heartbeat rate and behavior, NOR decreased feeding efficiency of *D. magna* even at low doses, therefore, it might seriously compromise ecosystem health and function.

Over the past decades, antibiotics have been widely used for both human and veterinary therapy. In China, the annual production of antibiotics exceeds 210,000 tons^1^, leading to an increased possibility of these antibiotics being released into aquatic environments. Norfloxacin (NOR) is one of the most widely used antibiotics since it is particularly efficient in treating diseases caused by both gram-negative and gram-positive bacteria. Thus, NOR residues can reach very high levels in hospital, animal production and aquaculture wastewaters, as well as in natural waters (e.g. rivers and lakes)^2^,^3^,^4^, which poses serious ecological risks.

Plankton has been widely used in studies examining the adverse effects (i.e., acute or chronic toxicity) of residual antibiotics on ecosystem functions^2^,^4^. These organisms occupy a significant position in the aquatic food chain, and are highly sensitive to environmental pollutants^4^,^5^. Given that NOR residues are difficult to biodegrade in aquatic ecosystems due to the lack of corresponding microbial decomposers^6^,^7^, these residues might exert persistent adverse effects on planktonic organisms. For example, previous studies have demonstrated that NOR alters the survival, growth, and physiology of the phytoplankton species *Scenedesmus obliquus* and *Chlorella vulgaris*^4^,^8^. Moreover, the growth of zooplankton individuals can be greatly inhibited by NOR^5^,^9^. Surprisingly, few studies have explicitly examined the role of NOR in phytoplankton-zooplankton interactions, which is required to understand the consequences of NOR contamination on ecological processes.

NOR may affect phytoplankton-zooplankton interactions (e.g., the feeding rate of grazers on algae) by inducing zooplankton behavioral responses to environmental changes. Zooplankton activity is highly sensitive to a wide range of environmental pollutants, including antibiotics, heavy metals, pesticides, and herbicides^10^,^11^. In response to environmental pollutants, zooplankton individuals may increase their swimming ability (i.e., increase velocity in horizontal or vertical movement, or improve three-dimensional movement by increasing swimming velocity and frequency in both horizontal and vertical movements)^12^,^13^,^14^, or decrease it^15^,^16^, depending on the species, dosage, exposure time and pollutant type^10^. In addition, the swimming pattern (e.g., the time and energy allocated to vertical and horizontal swimming than can greatly affect grazers swimming ability^17^) and the heartbeat rate of zooplankton individuals might also be modified when they are exposed to pollutants^18^,^19^.

Altering the swimming ability of zooplankton might further affect individual’s feeding rate if they swim to obtain food^20^,^21^. The mechanism might be of relevance to the affected encounter rate between predator and its prey, assuming that encounter rate is a function of the swimming ability and body size of both predator and prey species^17^. Many studies have reported alterations in feeding rates as a result of altered swimming abilities after exposure to pesticides and heavy metals^10^,^22^. Unfortunately, the impact and mechanism by which NOR might affect phytoplankton-zooplankton relationships is still largely unknown^2^.

The present study was designed to test if NOR affects phytoplankton-zooplankton interactions, based on the feeding rate of zooplankton on phytoplankton. Thus, we conducted four separate experiments to examine *Daphnia magna* lethal (mortality rate) and sublethal (heartbeat rate, behavior and feeding rate) responses to NOR in the absence or presence of its prey, alga *Chlorella pyrenoidosa. D. magna* is a keystone species in freshwater lakes and ponds, and *C. pyrenoidosa* is one of its preferred foods^17^. We hypothesized that: (1) mortality rate will gradually increase with increasing NOR concentration and exposure time, and (2) heartbeat rate, average swimming ability and feeding rate will change after NOR exposure; a positive correlation is also expected between average swimming ability and feeding rate.

## Results

### Lethal responses: mortality

**Figure 1:**
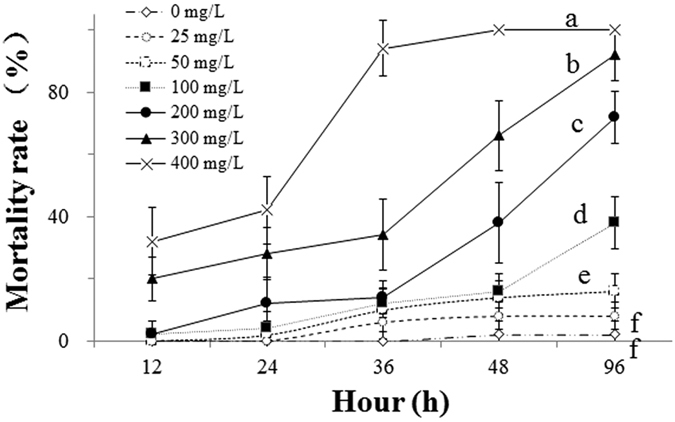
Mortality rates (means ± s.d., n = 5) of *Daphnia magna* individuals, at the 12^th^, 24^th^, 36^th^, 48^th^, and 96^th^ h after exposure to seven norfloxacin concentrations (0, 25, 50, 100, 200, 300, and 400 mg L^−1^). Different letters indicate significant differences (*P* < 0.05) among norfloxacin concentrations throughout the 96-h-long experiment, according to one-way repeated measures analysis of variance (rm-ANOVA). Figure 1 was produced in Microsoft Excel 2016.

The mortality rate of *Daphnia magna* was significantly affected by norfloxacin (NOR) concentration, exposure time, and their interactions in the absence of the green alga *Chlorella pyrenoidosa* (Table 1, Fig. 1, *P* < 0.001). According to repeated measures analysis of variance (rm-ANOVA), exposure to increasing NOR concentrations ranging from 0 to 400 mg L^−1^ led to increases in mortality rates throughout the 96-h long experiment, demonstrating dose dependency. Specifically, the control treatment cause minimal fatalities (<2%), and the 25 mg L^−1^ NOR treatment did not produce a significant increase in mortality rate compared to the control treatment (*P* > 0.05). In contrast, mortality rates significantly increased in 50, 100, 200, 300, and 400 mg L^−1^ NOR concentrations compared to the control treatment (Fig. 1; *P* < 0.05), reaching 16.0%, 38.0%, 72.0%, 92.0% and 100% at the end of the experiment, respectively. Additionally, at a given NOR concentration, mortality rate of *D. magna* generally increased with increasing exposure time, exhibiting a time-dependent acute toxicity. Correspondently, the 12-, 24-, 36-, 48- and 96-h LC_50_ were 561.9, 527.5, 295.6, 175.8 and 107.6 mg L^−1^, respectively (Table 2).

### Sublethal responses: heartbeat rate

**Figure 2:**
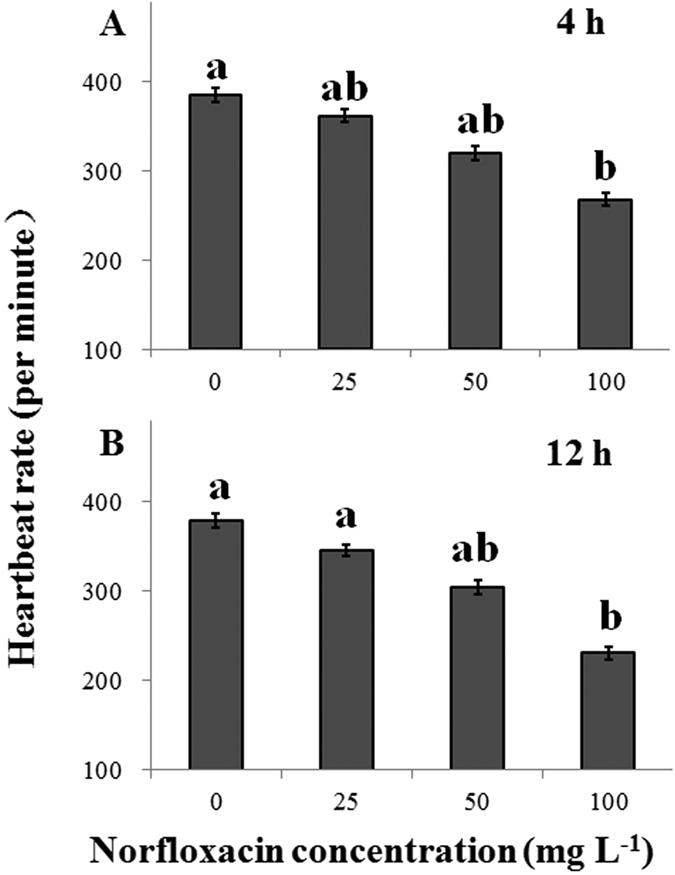
Heartbeat rates (means ± s.d., n = 5) of *Daphnia magna* individuals after 4 (**A**) and 12 h (**B**) of exposure to four norfloxacin concentrations (0, 25, 50 and 100 mg L^−1^). Differences between mean heartbeat rates at the 4^th^ and 12^th^ h of the experiment were assessed by one-way analysis of variance (ANOVA) and one-way analysis of covariance (ANCOVA), respectively, followed by Tukey’s *post hoc* tests. Different letters indicate significant differences among norfloxacin concentrations (*P* < 0.05). Figure 2 was produced in Microsoft Excel 2016.

Heartbeat of *D. magna* was greatly affected by NOR concentration in the presence of *C. pyrenoidosa* (*P* < 0.03; Fig. 2A and B). Along with increasing NOR concentration, heartbeat rate reduced by 6.0%, 16.7%, and 30.4% after 4 h of exposure, and by 8.8%, 19.5%, 39.0% after 12 h of exposure in NOR concentrations of 25, 50 and 100 mg L^−1^ compared to the control treatments.

### Sublethal responses: swimming behavior

**Figure 3:**
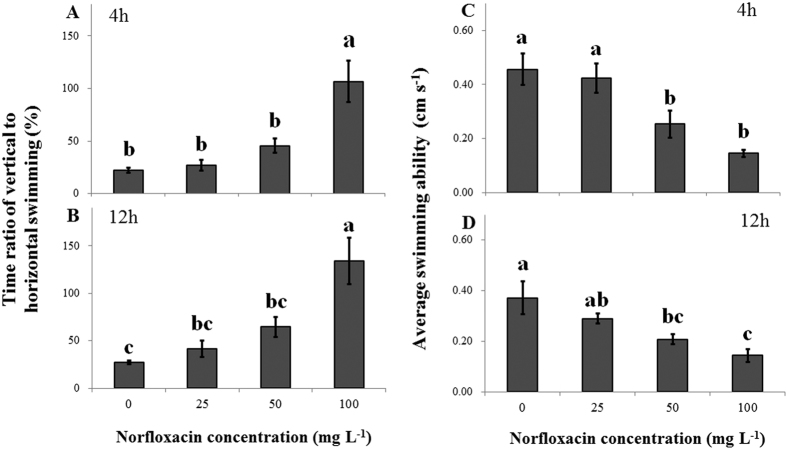
Time ratios of vertical to horizontal movement (**A**,**B**; means ± s.d., n = 5) and average swimming abilities (**C**,**D**; means ± s.d., n = 5) of *Daphnia magna* individuals after 4 (**A,C**) and 12 h (**B**,**D**) of exposure to four norfloxacin concentrations (0, 25, 50 and 100 mg L^−1^). Differences between the mean values of both variables at the 4^th^ and 12^th^ h of the experiment were assessed by one-way analysis of variance (ANOVA) and one-way analysis of covariance (ANCOVA), respectively, followed by Tukey’s *post hoc* test. Figure 3 was produced in Microsoft Excel 2016.

**Figure 4:**
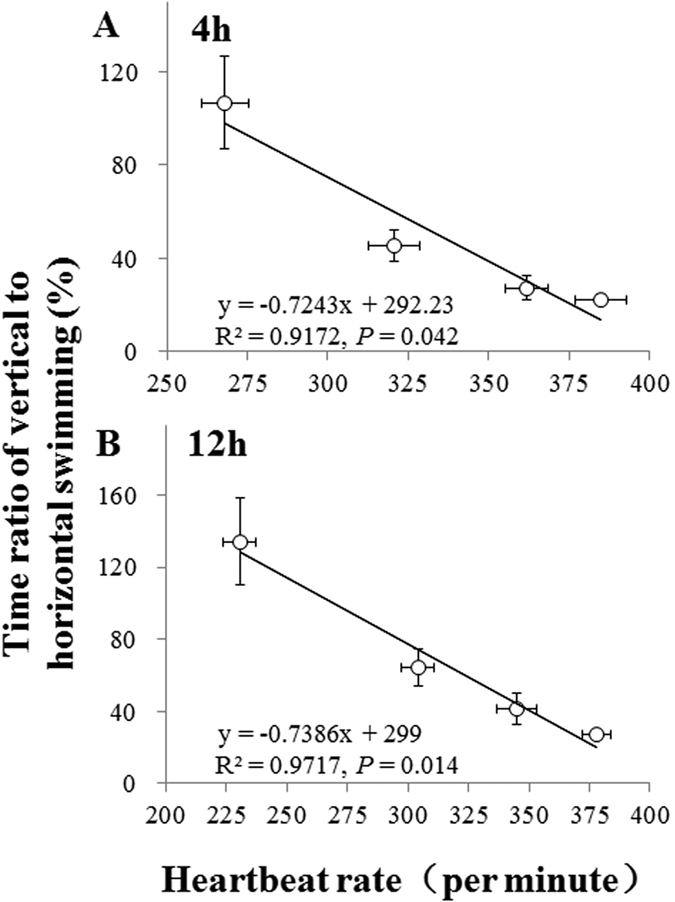
Relationships between heartbeat rate and time ratio of vertical to horizontal movement for *Daphnia magna* individuals after 4 (**A**) and 12 h (**B**) of exposure to four norfloxacin concentrations (0, 25, 50 and 100 mg L^−1^). Figure 4 was produced in Microsoft Excel 2016.

Swimming behavior in the presence of *C. pyrenoidosa* was greatly affected by NOR concentrations after 4 and 12 h of exposure (Table 3). Specifically, increasing NOR concentration led to a significant increase in the duration of quiescence and to a significant decrease in the duration of horizontal movement (*P* < 0.05; Table 3), with a correspondingly increase in the time ratio of vertical to horizontal swimming (TVH, *P* < 0.01; Fig. 3A and B), regardless of experimental time. Horizontal- and vertical-swimming velocities were not affected by NOR concentration (*P* > 0.05; Table 3). Collectively, increasing NOR concentrations of 25, 50 and 100 mg L^−1^ reduced average swimming ability by 7.3%, 44.5% and 68.1% after 4 h of exposure, and by 22.1%, 44.3% and 61.5% after 12 h of exposure, compared to the control treatments (Fig. 3C and D). Additionally, heartbeat rate and TVH (Fig. 4A and B) was significantly and negatively associated, regardless of experimental time (R^2^ > 0.9 in all linear regressions).

### Sublethal responses: feeding rate

**Figure 5:**
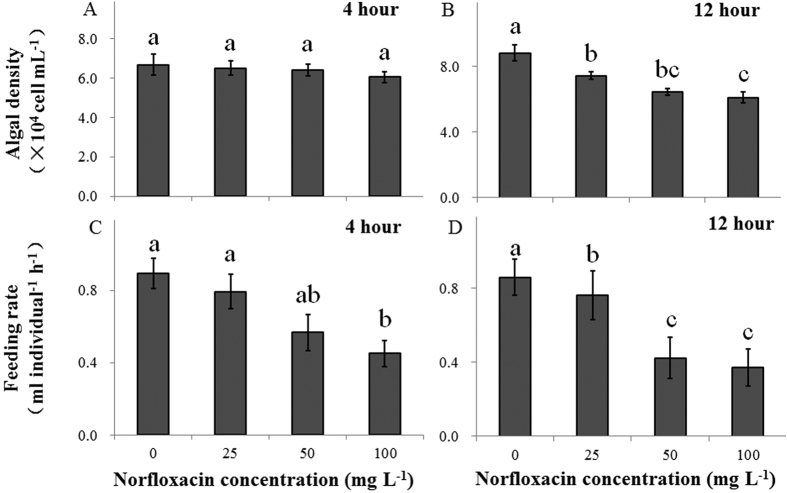
Density (**A**,**B**; means ± s.d., n = 5) of *Chlorella pyrenoidosa* in the absence of grazers and feeding rates (**C**,**D**; means ± s.d., n = 5) of *Daphnia magna* on *C. pyrenoidosa* after 4 (**A**,**C**) and 12 h (**B**,**D**) of exposure to four norfloxacin concentrations (0, 25, 50 and 100 mg L^−1^). Differences between algal density of *C. pyrenoidosa* rates at the 4^th^ and 12^th^ h of the experiment were assessed by one-way analysis of variance (ANOVA), followed by Tukey’s *post hoc* tests. Differences between mean feeding rates of *D. magna* at the 4^th^ and 12^th^ h of the experiment were measured by one-way ANOVA and one-way analysis of covariance (ANCOVA), respectively, followed by Tukey’s *post hoc* test. Figure 5 was produced in Microsoft Excel 2016.

**Figure 6:**
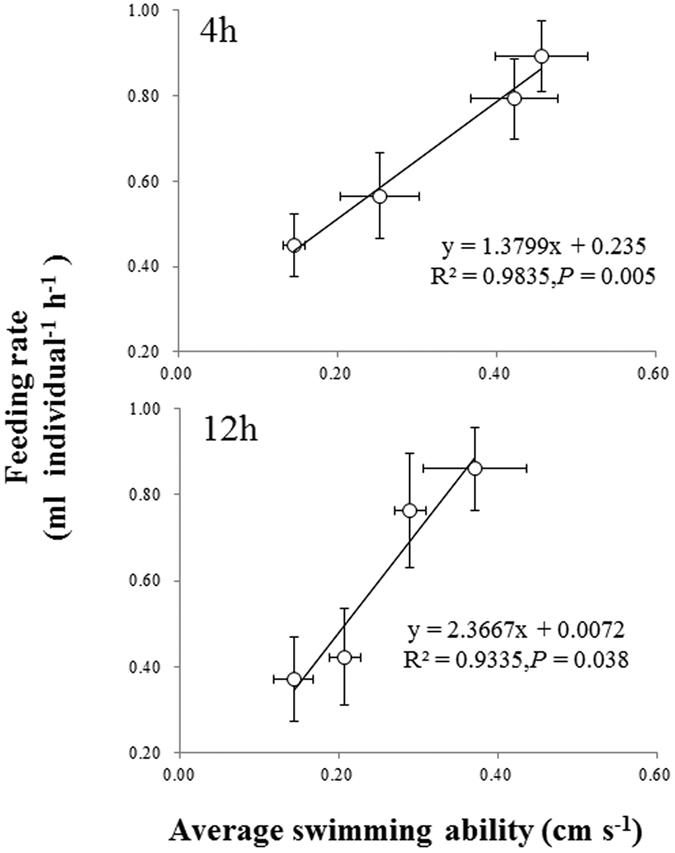
Relationships between the swimming ability and the feeding rate of *Daphnia magna* individuals after 4 (**A**) and 12 h (**B**) of exposure to four norfloxacin concentrations (0, 25, 50 and 100 mg L^−1^). Figure 6 was produced in Microsoft Excel 2016.

In the absence of grazers, *C. pyrenoidosa* density was not affected by NOR concentrations after 4 h of exposure (*P* > 0.05, Fig. 5A), but decreased significantly with increasing NOR concentrations after 12 h of exposure (*P* < 0.05, Fig. 5B). One-way analysis of variance (ANOVA) at the 4^th^ h and one-way analysis of covariance (ANCOVA) at the 12^th^ h showed that the feeding rate consistently decreased with increasing NOR concentrations (*P* < 0.01, Fig. 5C and D). Moreover, the feeding rate was positively associated with average swimming ability in this alga-grazer system, regardless of experimental time (Fig. 6, r^2^ > 0.90 in all regressions).

## Discussion

Although NOR is increasingly used in clinical treatments, its behavior and biological consequences when released into aquatic environments are still poorly understood. According to the results obtained in the present study, high NOR concentrations led to *D. magna* death, while low NOR concentrations affected individuals’ heartbeat rate and behavior, which further affected their feeding rate on *C. pyrenoidosa* (see Supplementary Fig. S1). The effects of NOR on the survival and growth of plankton species have been previously reported^4^,^23^, but our study is one of the few experimental assessments showing how NOR contamination affects phytoplankton-zooplankton interactions.

In our lethal experiment, higher NOR concentrations and longer exposure times increased mortality rates. Mortality rates significantly increased after 12 h of exposure in concentrations of 300 and 400 mg L^−1^, after 24 h of exposure in concentrations of 200, 300 and 400 mg L^−1^, and after 36 h of exposure in concentrations of 50, 100, 200, 300, and 400 mg L^−1^. After 96 h of exposure, mortality rate increased from less than 2% to 100% with increasing NOR concentration. The above-mentioned results support our first hypothesis that mortality rate would gradually increase with increasing NOR concentration and exposure time. Moreover, we found that the magnitude of the contamination was a more important determinant for NOR toxicity to *D. magna* individuals than exposure time (Table 1), similarly to that reported for toxicity of organic pollutants, such as fenvalerate^24^ and chlorpyrifos^25^.

In sublethal experiments, NOR contamination led to a significantly decrease in heartbeat rate of *D. magna*, indicating that NOR can seriously compromise heart function. Heartbeat rate has been used as an indicator of *D. magna* physiological responses in chemical exposure studies, and toxicant-induced changes in heartbeat rate can lead to long-term effects on its ecological processes, such as individual behavior and population dynamics^19^,^26^. Research shows that the disorder of heartbeat rate often means haematological changes and organ damages that would reduce respiratory capacity while increase respiratory demand and oxygen consumption under contaminated circumstances^19^. As a result, *D. magna* individuals have to move to the water surface to maximize oxygen uptake, which affects their swimming pattern, as indicated by the increased TVH obtained in the present study, which was significantly and negatively correlated with heartbeat rate (Fig. 4A and B).

Vertical swimming represents an additional energy cost in relation to horizontal swimming as *D. magna* individuals have to overcome both viscous drag and gravity when they swim upward^27^,^28^. Besides, there are trade-offs between the adaptation to specific stress and movement, such as increasing the tolerance to a specific stressor through maintaining movement at a low level^17^,^29^. Consequently, the duration of quiescence significantly increased with increasing TVH, which finally led to a decrease in average swimming ability. Numerous studies have identified decreases in swimming ability of zooplankton individuals when exposed to environmental contaminants. For example, Untersteiner *et al*.^16^ reported a decreased swimming ability in *D. magna* as a result of copper exposure, and Artells *et al*.^30^ found a low swimming ability in *Daphnia similis* and *Daphnia pulex* exposed to cerium dioxide nanoparticles.

Exposure to NOR might further affect the feeding rate of *D. magna*, which is often positively correlated with swimming ability^17^,^20^,^21^. Most studies indicated that pollutant exposure reduce the feeding behavior of cladocerans^26^,^31^, because pollutants can inhibit swimming ability and ingestion efficiency of zooplankton species^32^,^33^. Demott (1991)^34^ suggested that feeding inhibition is either a very important adaptation to pollutants by avoiding ingesting toxins, or a direct consequence of poisoning. Our results suggested that reduced feeding rate of *D. magna* might be a direct consequence of NOR poisoning, i.e., of the swimming inhibition due to NOR contamination, as there was a significant positive relationship between average swimming ability and feeding rate (Fig. 6). These results provide supports for our second hypothesis of a positive relationship between average swimming ability and feeding rate in individuals exposed to NOR.

In summary, the present study revealed that NOR greatly affects the biological functions of zooplankton individuals, and the corresponding phytoplankton-zooplankton relationships. Our results might have important implications for understanding the ecological consequences of NOR accumulation in plankton systems. Moreover, altered the grazer swimming activity and thus feeding rate may affect population dynamics of grazer species due to the close relationships between the survival, growth and reproduction with feeding rate^35^. Additionally, as a result of the behavioral inhibition caused by NOR, the encounter rates between *D. magna* individuals and their predator might be reduced, but, when they encountered, the attack success of the predator on these grazers would be stimulated, which will definitely affect predator-prey dynamics^17^,^36^. Thus, further research on the potential effects of NOR on population dynamics, predator-prey interactions, and community structure should be developed.

Noteworthy, the NOR concentrations used in our sublethal experiments were slightly higher than that used in studies examining other environmental contaminants^34^, because, according to the results of our lethal experiment and to previous studies^4^,^8^, NOR presents low toxicity to plankton organisms. However, this does not reduce the universal and important ecological risks imposed by NOR contamination, given that its concentration has already reached a high level (of several μg to mg per litre) in some aquatic environments^2^,^3^,^4^, and there is no doubt that this problem will become even more severe in the future.

## Materials and Methods

### Experimental organisms

The ecological effects of the antibiotic norfloxacin (NOR) on aquatic ecosystems were determined using the phytoplankton species *Chlorella pyrenoidosa* and the zooplankton species *Daphnia magna*, which are commonly used in standardized toxicity tests^37^. This research was conducted under Law of the People’s Republic of China on the Protection of Wildlife (August 28, 2004). No permits were required to carry out this study. All animal work was approved by the Animal Care Committee at Yunnan University.

Samples of *C. pyrenoidosa* (FACHB-28) were obtained from the Institute of Hydrobiology, a part of the Chinese Academy of Sciences. Algae were batch cultured (600 mL in 1000-mL Erlenmeyer flasks) axenically in liquid COMBO medium at 25 °C with a light: dark cycle of 14 h:10 h and a light intensity of 120 μmol photons m^−2^ s^−1^ in a light incubator (LRH-400-GSI; Shaoguan Thaihung Medical Instruments, Shaoguan, China).

The *D. magna* individuals used in this study (in all four independent experiments) were derived from Dianchi Lake (Kunming City, Yunnan Province, China). Stock populations were reared at 25 °C and 14 h:10 h light: dark cycle in COMBO medium, and fed with *C. pyrenoidosa* at a rate of 10^5^ cells mL^−1^ day^−1^ for three months prior to the experiment. Before starting the experiments, grazers were transferred to clean COMBO medium and starved for at least 2 h to empty their guts. All experiments were performed with neonates (about two days old) in order to avoid maternal influence because large individuals could contain embryos and reproduce during the experiments, as reported in previous studies^38^,^39^,^40^,^41^.

### Experimental design

All laboratory experiments were conducted at Yunnan University, Kunming, China, starting on August 4, 2015. The NOR purchased from Dalian Meilun Biology Technology Company, Ltd. (Liaoning, China). NOR stock solution was first dissolved in 1 M NaOH to yield a 1,000 mg L^−1^ solution, neutralized with 1 M HCl, and then diluted in COMBO medium to obtain the designed concentrations according to Nie *et al*.^4^. Two additional experiments included only NOR solubilizers (containing both NaOH and HCl as the NOR solutions, but without adding NOR) showed that these two components had no significant influence on heartbeat rate and average swimming ability of *D. magna* individuals after 12 h of exposure compared to the control treatments (in the absence these two components), so their effects on plankton systems were considered negligible (see Supplementary Table S1).

Four separate experiments were conducted to investigate the NOR lethal toxicity (in the absence of *C. pyrenoidosa*) on *D. magna* mortality rate, and sublethal toxicity (in the presence of *C. pyrenoidosa*) based on *D. magna* heartbeat rate, behavior and feeding rate.

In the lethal experiment, *D. magna* individuals were exposed to seven NOR concentrations (0, 25, 50, 100, 200, 300 and 400 mg L^−1^) for 12, 24, 36, 48 and 96 h. Each treatment had five replicates. The NOR concentration range used here was based on the 48-h LC_50_ value of about 200 mg L^−1^ as indicated by Liu *et al*.^23^.

In three sublethal experiments, four NOR concentrations (0, 25, 50, and 100 mg L^−1^) were used to evaluate *D. magna* heartbeat rate, activities and feeding rate, in the presence of *C. pyrenoidosa* after 4 and 12 h of exposure, respectively. The NOR concentrations tested were selected based on the results obtained in the lethal experiment (i.e., on the NOR treatments that had no significant effects on mortality rate of *D. magna* after 12 h of exposure), and were within the range used in previous sublethal studies addressing zooplankton activity and phytoplankton-zooplankton interactions^5^,^9^. Each test had five replicates. Meanwhile, an additional system containing only algal species was also set up for each of the above NOR concentrations (each with 5 replicates) in the feeding experiment so as to accurately estimate the feeding rate of *D. magna* later.

Experiments containing *D. magna* individuals were conducted in 500 mL beakers containing 200 mL of medium and the tested NOR concentration, on which *D. magna* individuals (with density of 50 individuals L^−1^) were introduced, followed by inoculation of *C. pyrenoidosa* cells (with density of 5 × 10^4^ cells mL^−1^). The densities of both predators and preys were close to those used in previous studies considering species interactions and grazer activities^17^.

To homogenize oxygen contents among beakers, these were gently aerated with sterile filtered air (Sartorius, Midisart 2000) and mechanically stirred at 60 rpm using an incubation shaker (THZ-103B, Shanghai Yiheng Scientific Instrument Co. Ltd., Shanghai, China) for 10 min, before starting the experiments. After stirring, beakers used in the mortality, heartbeat rate and feeding rate experiments were transferred to incubators (LRH-400-GSI; Shanghai Yinze Instrument Equipment, Shanghai, China) set at 25 °C, and using approximately 120 μmol photons m^−2^ s^−1^ light intensity, while, beakers used in the behavioral experiment were placed in an observation platform.

### 96-h-long lethal experiment

The 96-h-long lethal experiment conducted in the present study followed the OECD Guideline 202, with slight modifications related to water-quality determination of substances’ acute toxicity for *Daphnia* spp. (e.g. *Daphnia magna*), and the national standards (China, GB/T 13266–91). Ten randomly selected neonates were placed in 100 mL glass beakers containing 30 mL of the NOR test solution. Thus, the grazer density used here (3 individuals mL^−1^) was similar to that used in previous studies on acute toxicity^39^. *D. magna* individuals were not fed during the test periods following previous studies^39^,^42^,^43^.

During experiments, all beakers were removed from the light incubator every hour and mechanically stirred at 80 rpm for 10 minutes in an incubation shaker (THZ-103B, Shanghai Yiheng Scientific Instrument Company. Ltd., Shanghai, China) to facilitate gas exchange.

### Sublethal experiment: heartbeat

At the 4^th^ and 12^th^ h after exposure to the four NOR concentrations, one *D. magna* individual was randomly selected from each beaker and transferred to an 8 mL transparent cuboid polyethylene chamber containing 3 mL of medium and NOR concentrations corresponding to those they had been exposed to, and observed under an Olympus BX51microscope with a DP73 imaging system (Olympus, Japan). Each *D. magna* was tethered to a hair using glue (3 M, Animal Care Products, St. Paul, MN, USA) following Lovern *et al*.^19^. They were allowed to acclimate to the experimental conditions for 10 min before recordings, which lasted for 1 min. After measurements, each *D. magna* was returned to the original beaker and subjected to further incubation.

### Sublethal experiment: behavioral

We used a two-camera filming system to quantify *D. magna* swimming behavior. The two video cameras (FDR-AX30, Sony, Japan; spatial resolution: 3840 × 2160 pixels) were placed orthogonally at 20 cm from the projective plane of the beaker (i.e., the bottom or the backside of the beaker) to record the swimming activity of a grazer individual in both horizontal and vertical directions simultaneously. Standard fluorescent bulbs were installed at approximately 2 m around the experimental setup (providing 40 μmol photons m^−2^ s^−1^ illumination) to minimize the effect of positive phototaxis on individuals’ behaviors. *D. magna* individuals were allowed to acclimatize to the new environment for 15 min before the initiation of recording. Each recording comprised two 5-min sequences, one in the horizontal direction and one in the vertical direction, and was taken at 50 frames s^−1^.

Video recordings were reviewed in Adobe After Effects CS4 (Adobe, USA) as described by Pan *et al*.^17^. Four different behaviors were observed for each randomly-chosen grazer: horizontal swimming, vertical upward swimming, vertical downward swimming, and quiescent status. Firstly, we recorded the time spent on each behavior. Then we selected the video fragments displaying swimming trajectories away from the walls of the beaker (i.e., in the middle of both camera views) and that lasted longer than 2 s, to calculated instantaneous swimming velocity as the distance swum by this grazer individual between two frames (i.e. 20.4 ms) using ImageJ 1.46 and MTrackJ plugin following Moison *et al*.^41^. While analyzing the swimming velocity of an individual, each video was first calibrated to convert pixels into real distances (mm) using reference that were surrounded of the beaker. Finally, the average swimming ability (*V*_*average*_, mm s^−1^) of each individual was calculated as:


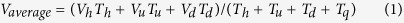


where *T*_*h*_, *T*_*u*_, *T*_*d*_ and *T*_*q*_ were the durations of horizontal, upward, and downward swimming, and quiescence (s), respectively; *V*_*h*_, *V*_*u*_, and *V*_*d*_ were the velocities of horizontal, upward, and downward swimming (mm s^−1^), respectively.

Three grazers were randomly chosen to determine the above-mentioned metrics in each beaker, and thus a total of 60 grazers (3 individuals per beaker × 5 beakers per treatment × 4 treatments) were analysed at each experimental time. The values obtained were first averaged for each beaker and then for each treatment.

### Sublethal experiment: feeding rate

To study the effects of NOR on feeding rate of *D. magna*, algae were sampled from each beaker after 4 and 12 h of exposure to four NOR concentrations. At the end of the experiment, algal densities in all treatments were greater than 4.5 × 10^4^ cells mL^−1^, indicating that *D. magna* individuals were unlikely to be food-limited throughout the feeding experiment^44^.

To count *C. pyrenoidosa* cells, 2 mL of the solution contained within each experimental beaker were transferred to a 10 mL tube containing 0.1 mL Lugol’s preservative. Algal density was directly determined using a fluorescence microscope (Olympus BX51 with a DP73 imaging system; Olympus, Japan) at ×400 magnification. The feeding rate (*FR*, mL animal individual^−1^ h^−1^, i.e., clearance rate) was calculated as the difference between algal density in experimental treatments (containing grazers) and the corresponding controls (without grazers; *C. pyrenoidosa* only) according to the following equation, commonly used in plankton^45^:


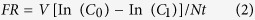


where *V* is the volume of the culture (mL); *C*_*0*_ and *C*_*1*_ are the algal density at the end of the experiments in the control and experimental beakers (cells mL^−1^), respectively; *N* is the number of *D. magna* and *t* is the duration of the experiment (h).

### Data analysis

All data were tested for normality and variance heterogeneity before analyses. The 12-, 24-, 36-, 48- and 96-h LC_50_ and their associated 95% confidence intervals (95% CI) were calculated by the probit analysis method. One-way analysis of variance (ANOVA) was used to determine the effect of NOR concentration on heartbeat rate, swimming behavior, and average swimming ability, as well as feeding rate of *D. magna* after 4 h of exposure; these were followed by a Tukey’s *post hoc* test once a significant effect was detected. While, one-way analysis of covariance (ANCOVA) followed by Tukey’s *post hoc* test was used to determine the effect of NOR concentration on the above-mentioned variables after 12 h of exposure (using algal density in the absence of grazers as the covariate). One-way repeated measures analysis of variance (rm-ANOVA) was used to test the effect of NOR concentration on *D. magna* mortality rate. The Greenhouse–Geisser instead of the sphericity assumption was applied to recalculate the *F*-value because of violation following previous studies^35^. In addition, linear regression analyses were conducted to determine the relationships between heartbeat rate and time ratio of vertical to horizontal movement (TVH), and between the average swimming ability and feeding rate. All analyses were carried out using IBM SPSS19.0 package (SPSS Inc., USA).

## Additional Information

**How to cite this article**: Pan, Y. *et al*. Lethal/sublethal responses of *Daphnia magna* to acute norfloxacin contamination and changes in phytoplankton-zooplankton interactions induced by this antibiotic. *Sci. Rep.* **7**, 40385; doi: 10.1038/srep40385 (2017).

**Publisher's note:** Springer Nature remains neutral with regard to jurisdictional claims in published maps and institutional affiliations.

## Supplementary Material

Supplementary Material

## Figures and Tables

**Table 1:** One-way repeated measures ANOVA result of the effects of time and norfloxacin concentration on mortality rate of *Daphnia magna*.

	Time		df	Concentration (C)		df	Time × C		df
	*F*	*P*		*F*	*P*			*F*		*P*
Mortality rate (%)	187.157	<0.001	4	898.460	<0.001	6	19.546	<0.001	24

**Table 2:** The 12-h, 24-h, 36-h, 48-h, and 96-h LC_50_ values and their associated 95% confidence intervals (95% CI) of *Daphnia magna* when exposed to seven norfloxacin concentrations (0, 25, 50, 100, 200, 300 and 400 mg L^−1^).

	12-h	24-h	36-h	48-h	96-h
LC_50_ value (mg/L)	561.9	527.5	295.6	175.8	107.6
95% CI	441.5–1005.2	405.4–854.2	214.7–487.8	142.4–221.2	92.4–124.5

**Table 3:** Swimming activities (means ± s.d., n = 5) of *Daphnia magna* under four norfloxacin concentrations (0, 25, 50 and 100 mg L^−1^).

Exposure time	Treatment	Time distribution (s)				Swimming velocity (cm/s)		
		Quiescent status	Horizontal move	Upward swimming	Downward swimming	Horizontal move	Upward swimming	Downward swimming
4 hour	0 mg L^−1^	87.6 ± 46.0^c^	188.4 ± 38.0^a^	20.6 ± 7.9^a^	21.6 ± 6.5^a^	0.63 ± 0.08^a^	0.47 ± 0.08^a^	0.70 ± 0.27^a^
	25 mg L^−1^	118.4 ± 20.6^c^	155.6 ± 14.5^a^	22.2 ± 8.2^a^	19.8 ± 9.5^a^	0.71 ± 0.22^a^	0.34 ± 0.12^a^	0.66 ± 0.27^a^
	50 mg L^−1^	180.8 ± 21.2^b^	107.4 ± 21.5^b^	22.6 ± 5.9^a^	24.8 ± 7.9_a_	0.60 ± 0.19^a^	0.30 ± 0.07^a^	0.52 ± 0.30^a^
	100 mg L^−1^	238.6 ± 21.0^a^	56.6 ± 17.9^c^	31.0 ± 11.3^a^	26.2 ± 7.7^a^	0.50 ± 0.12^a^	0.27 ± 0.14^a^	0.54 ± 0.24^a^
12 hour	0 mg L^−1^	61.4 ± 38.8^c^	202.6 ± 17.5^a^	28.4 ± 11.7^a^	26.6 ± 7.6^a^	0.44 ± 0.16^a^	0.44 ± 0.23^a^	0.55 ± 0.15^a^
	25 mg L^−1^	128.0 ± 35.0^b^	148.0 ± 19.0^b^	29.8 ± 16.7^a^	30.2 ± 13.7^a^	0.49 ± 0.10^a^	0.49 ± 0.34^a^	0.41 ± 0.13^a^
	50 mg L^−1^	196.6 ± 19.7^a^	99.4 ± 13.1^c^	30.4 ± 7.4^a^	31.6 ± 10.2^a^	0.49 ± 0.19^a^	0.37 ± 0.07^a^	0.44 ± 0.30^a^
	100 mg L^−1^	220.6 ± 12.3^a^	50.2 ± 13.5^d^	32.6 ± 15.7^a^	34.2 ± 21.0^a^	0.43 ± 0.15^a^	0.49 ± 0.28^a^	0.41 ± 0.21^a^

Different letters indicate significant differences among the four norfloxacin concentrations (*P* < 0.05).
