# Supplementary material for: Lethal/sublethal responses of Daphnia magna to acute norfloxacin contamination and changes in phytoplankton-zooplankton interactions induced by this antibiotic
Source: Sci Rep. 2017 Jan 12;7:40385. doi: 10.1038/srep40385 (PMC5227989; doi:10.1038/srep40385)
Supplement: Supplementary Material [file srep40385-s1.pdf]

Lethal/sublethal responses of *Daphnia magna* to acute norfloxacin contamination and changes in phytoplankton-zooplankton interactions induced by this antibiotic

Ying Pan[1†], Shi-wei Yan[1†], Ruozhu Li[1], Yi-wen Hu[2], Xue-xiu Chang[1*]

[1]School of Ecology and Environmental Sciences, Yunnan University, Kunming 650091, China

[2]Department of Biology, Yunnan University, Kunming 650091, China

[†] These authors contributed equally to this work

[*] Corresponding author: E-mail: changxx@ynu.edu.cn

Tel: +86 871 5033547; fax: +86 871 5033547

Supplementary Fig. S1 A conceptual figure of the effects of norfloxacin concentration in affecting phytoplankton-zooplankton interactions. This figure was produced in Microsoft PowerPoint 2016.

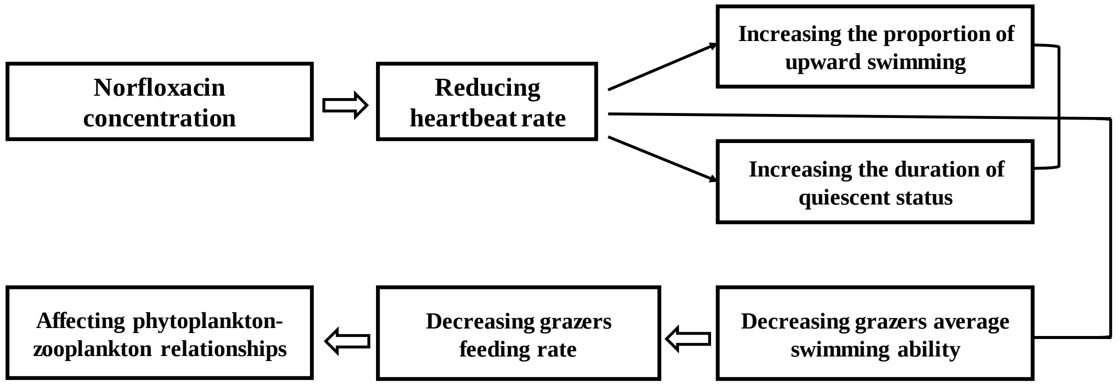

Supplementary Table S1 Heartbeat rate and average swimming ability of *Daphnia magna* individuals under different solutions (containing both NaOH and HCl as the norfloxacin solutions, but without adding norfloxacin) after 12 h of exposure cultivation. Different letters indicate significant differences among treatments. Multiple comparisons of means were performed using Tukey's test at the 0.05 significance level.

Concentration of two solubilizers	n	Heartbeat rate (per minute)	Average swimming ability (mm s^{-1})
Without two solubilizers	5	394.0±11.9[a]	0.36±0.10[a]
As the norfloxacin concentration of 25 mg L^{-1}	5	398.8±17.1[a]	0.37±0.06[a]
As the norfloxacin concentration of 50 mg L^{-1}	5	389.0±13.6[a]	0.34±0.11[a]
As the norfloxacin concentration of 100 mg L^{-1}	5	385.0±27.0[a]	0.31±0.05[a]
